# The toll like receptor 7 pathway and the sex bias of systemic lupus erythematosus

**DOI:** 10.3389/fimmu.2025.1479814

**Published:** 2025-02-20

**Authors:** R. Hal Scofield, Jonathan D. Wren, Valerie M. Lewis

**Affiliations:** ^1^ Arthritis & Clinical Immunology Program, Oklahoma Medical Research Foundation, Oklahoma City, OK, United States; ^2^ Department of Medicine, College of Medicine, University of Oklahoma Health Sciences Center, Oklahoma City, OK, United States; ^3^ Research and Medical Services, Oklahoma City US Department of Veterans Health Care System, Oklahoma City, OK, United States; ^4^ Genes and Human Disease Research Program, Oklahoma Medical Research Foundation, Oklahoma City, OK, United States

**Keywords:** systemic lupus erythematosus, sex bias, TLR7, TASL, XIST

## Abstract

Systemic lupus erythematosus (SLE) predominately affects women with a ratio of females-to-males of about 9:1. The complement of sex chromosomes may play and important role in the mechanism of the sex bias. Previous work has shown that men with Klinefleter’s syndrome (47,XXY) as well as women with 47,XXX are found in excess among SLE patients well as among Sjogren’s disease, systemic sclerosis and idiopathic inflammatory myositis. in cells with more than one X chromosome, all but one is inactivated. However, X chromosome inactivation, as mediated by the long noncoding RNA X-inactive specific transcript, or XIST, is not complete with approximately 10% of genes in the non-recombining region of the X chromosome escaping X inactivation. In the TLR7 signaling pathway, both the TLR7 and TLR adaptor interacting with endolysosomal SLC15A4 (TASL) escape X inactivation. Comparing male and female immune cells, there is increased TLR7 signaling related to increased expression of these genes in cells with more than one X chromosome. Cells with more than one X chromosome also express XIST, while cells with one X chromosome do not. XIST, as a source of ligand for TLR7, has also been shown to increase TLR7 signaling. Thus, we propose that both these mechanisms operating in immune cells with more than one X chromosome may act in a mutual way to mediate an X chromosome dose effect for the sex bias of autoimmune disease.

## Sex bias in lupus

Systemic illness among patients with the rash of lupus erythematosus was first noted by Moriz Kaposi in Vienna during the late 19^th^ century (1). During the remainder of the 19^th^ century and through the middle of the 20th century, the entity of systemic lupus erythematosus was established (2). The bias of this disease to affect women was also noted during this period, with assembled cohorts comprised by ~90% of women (3). This ratio of ~9:1 women to men in cohorts of SLE has continued to be true into the 21^st^ century with modern epidemiological methods (4). This relationship holds true in all racial and ethnic groups studied.

## Sex hormones in lupus

While there are sex hormone differences between SLE patients and matched controls, be they men or women (reviewed in (5)), a fundamental biological explanation for these findings and their relationship to the gender-bias of SLE has not been forthcoming (6). Clearly, some men with SLE have primary hypogonadism. For instance, Mok, et al, found that 5 of 35 men with SLE had low serum testosterone and high luteinizing hormone (LH) while none of 33 control men did (7). The etiology of the hypogonadism in these men was not determined. Higher serum prolactin is also found in both men and women with SLE compared to controls (8, 9). However, men with SLE have the same degree of hypogonadism and low testosterone as do men with other non-female biased chronic illnesses (10), suggesting chronic illness causes hypogonadism in SLE rather than vice versa. Furthermore, at the onset of disease, prior to treatment, there are no sex hormone differences between SLE patients and a matched control population (11).

## X chromosome in lupus

Seeking another explanation to the sex bias of SLE, we examined the complement of sex chromosomes, initially among men with SLE. We found that these SLE-affected men were much more likely than matched control men to have Klinefelter’s syndrome, that is, 47,XXY (12). Subsequent work found that 47,XXX was found in excess among women with SLE (13). We have also found the rare mosaic, 45XO/46XX/47XXX, is associated with SLE (14), while Turner’s syndrome (female 45,XO) was not found in excess among SLE patients (15). We have now extended these findings to other female-biased autoimmune diseases (16, 17), and others have replicated the findings in SLE (18, 19). Thus, this work established that the number of X chromosomes was a risk factor for SLE, and that the number of X chromosomes might underly the female predominance of the disease.

Discussing the potential mechanisms by which an X chromosome dose effect might operate requires a brief review of the biology of the sex chromosomes, which are in mammals, of course, are the X and Y. The X and Y chromosomes pair in meiosis and mitosis by virtue of short regions at the distal ends of both chromosomes known as the pseudoautosomal regions (PAR); namely PAR1 and PAR2. Each PAR contains a handful of genes, which behave identically to autosomal genes. That is, there is expression of one copy on X and one copy on Y with genetic crossover occurring within PAR1 and PAR2 of the X and Y chromosomes. Meanwhile, on the X chromosome, centromeric to the two PARs are about 2000 genes that are X-linked. Similarly, on the Y chromosome centromeric to the two PARs are about 40 genes in the non-recombining region of Y. Almost all these Y genes are expressed in male gonadal tissue and function in spermatogenesis. In contrast, X-linked genes, like other chromosomes, are not functionally organized; and, generally do not have a Y homologue (although there are exceptions).

**Figure 1:**
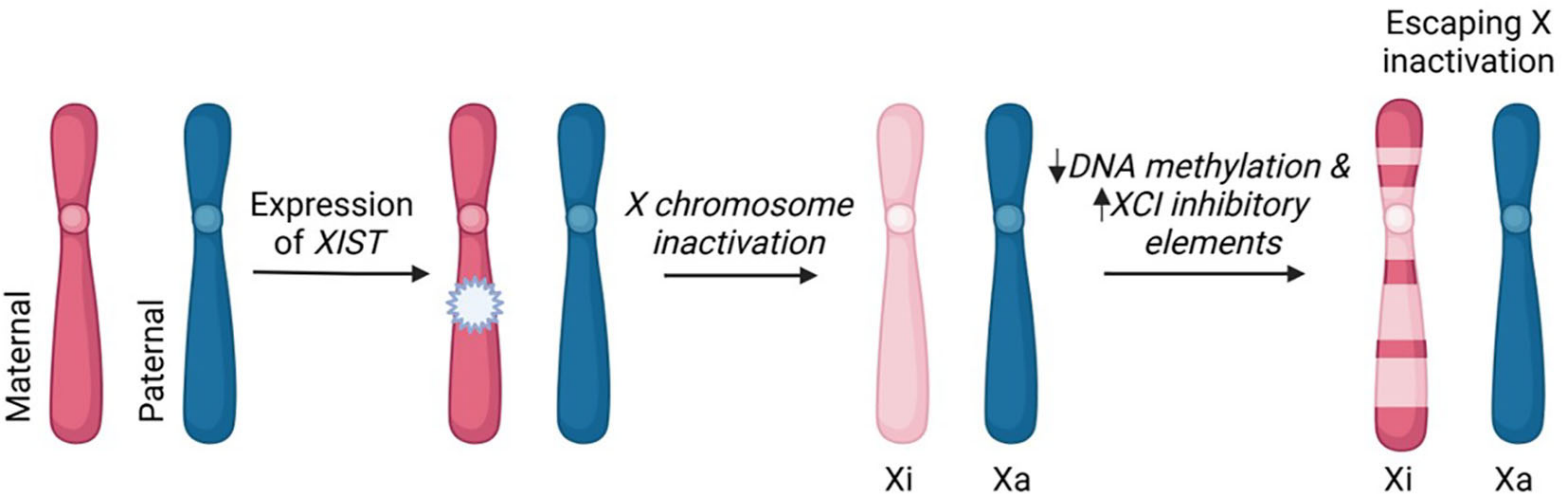
X chromosome inactivation and escaping X inactivation. The process of XCI occurs in mammalian cells that have two or more X chromosomes. In early stages of embryonic development, the maternal or paternal X chromosome is randomly silenced. This X-inactivation is initiated by long non-coding RNA, *XIST*, and subsequent DNA methylation and histone modifications. The incomplete inactivation of the X chromosome (pseudoautosomal region and variable genes throughout the X chromosome) results in approximately 15% of X-linked genes remaining transcriptionally active. These "escapee" genes contribute to differential expression of X-linked genes between men and women. Xi-inactive; Xa-active.This image was created in Biorender.com.

In cells with 2 or more X chromosomes, all but one is inactivated by methylation through the action of the X inactive-specific transcript (*Xist)* gene (**Figure 1**), which encodes a long non-coding RNA (20). That is, since women have two X-chromosomes and men have one, the imbalance in X chromosome gene expression is equalized by each cell with 2 or more X chromosomes randomly undergoing inactivation (which is mediated by methylation of CpG) of all but one X chromosome. However, despite the fact that the inactive X chromosome makes up the cytoplasmic Barr body, X inactivation is not an all-or-none phenomenon. On the inactivated X chromosome (X_i_), about 15% of the genes escape methylation partially or completely giving women (and Klinefelter men) more phenotypic variability compared to normal (i.e., 46XY) men (21).

Continued presence of *Xist* transcripts were not thought to be needed for maintenance of X inactivation (22). However, recent data demonstrate that this may not be the case in immune cells. Yu and colleague showed that deletion of Xist in CD11c-positive atypical memory B lymphocytes along with TLR7 activation induced isotype switching. In addition, *Xist* down regulation was found among B cells from women with SLE (23). Also, Anguera has found different localization patterns of the Xist non-coding RNA in B cells with upregulation of 20 X chromosome genes in female cells (24, 25). In a published preprint, conditional knock of Xist in female mice (BALB/c and C57BL/6) produced a spontaneous lupus phenotype (26). Thus, there may be differences in the physiology of this long non-coding RNA in B cells that change X chromosome inactivation in such a way that predisposes to a SLE.

## X chromosome and immune genes

The idea that immune genes are enriched on the X chromosome is frequently evoked. However, we find this is not the case. All human genes and Gene Ontology (GO) categories were downloaded from NCBI’s FTP server (ftp.ncbi.nlm.nih.gov/Gene/DATA/) on August 6^th^, 2024. Only protein-coding and RNA-producing (eg, ncRNA) transcripts with at least one GO category annotation were selected for analysis. GO categories associated with all transcripts on each human chromosome were then identified, summed, and hypergeometric tests performed to determine relative chromosomal enrichments or depletions in each GO category. False Discovery Rate (FDR) corrections for the most significant p-value (enriched or depleted) were performed to correct for multiple testing. As a positive control, we find the Y chromosome highly enriched (p-value = 0) in the GO categories “spermatogenesis” and “gonadal mesoderm development”. We find that, although there are many immune-related genes on the X chromosome, it is not particularly enriched for immune-related genes more than any other chromosome. This was true for all genes related to immune function with 50 of 1,482 (3.4%) on the X chromosome. Furthermore, no individual category of immune function had enrichment on the X chromosome (**Table 1**). In fact, we found significant immune-related transcript enrichment on other chromosomes, particularly chromosome 9 (**Table 2**), and we found other GO categories enriched on the X chromosome (**Supplementary Table S1**). Some of the categories in **Supplementary Table S1** might impact immune processes (eg, miRNA-mediated gene silencing), but none are not immune-specific.

**Table 1:** Immune related gene categories for the X chromosome.

GO group name/ID	on X/total	OR	FDR p value
innate immune response/0045087	14/485	0.81	0.6494
immune response/0006955	8/310	0.72	0.6494
adaptive immune response/0002250	4/193	0.58	0.6494
AHIRMAP/0061844	2/99	0.56	0.6494
activation of innate immune response/0002218	2/32	1.82	0.6494
positive regulation of innate immune response/0045089	2/30	1.95	0.6494
positive regulation of Ig production/0002639	2/28	2.10	0.6494
immunoglobulin mediated immune response/0016064	2/24	2.48	0.6494
immunological synapse formation/0001771	1/13	2.28	0.6494
negative regulation of immune response/0050777	1/12	2.48	0.6494
negative regulation of Ig production/0002638	1/8	3.90	0.6494
positive regulation of adaptive immune response/0002821	1/8	3.90	0.6494
regulation of immunoglobulin production/0002637	1/7	4.56	0.6494
T cell mediated immunity/0002456	1/16	1.82	0.6497
regulation of innate immune response/0045088	1/21	1.37	0.6521
regulation of immune system process/0002682	1/39	0.72	0.6585
innate immune response in mucosa/0002227	1/27	1.05	0.6617
regulation of immune response/0050776	1/27	1.05	0.6617
immune response-regulating signaling pathway/0002764	1/37	0.76	0.6625
humoral immune response/0006959	2/60	0.94	0.6642
positive regulation of immune response/0050778	1/36	0.78	0.6656

AHIRMAP, antimicrobial humoral immune response mediated by antimicrobial peptide.

**Table 2:** Gene ontology categories that are significantly found increased on a given chromosome.

chromosome	GO category/ID	#/total	OR	FDR p value	
9	0002286	TCA	17/24	57.99	0
9	0002323	NKCA	17/19	202.96	0
19	0002764	IRRSP	35/37	258.04	0
9	0006959	HIR	18/60	10.23	1.40E-08
6	0050778	PRIR	16/36	13.54	2.15E-08
19	0002682	RISP	15/39	9.21	4.00E-06
20	0045087	IIR	36/485	2.83	6.69E-05
9	0002250	AIR	25/193	3.56	0.0001
6	0002250	AIR	30/193	3.12	0.0001
4	0061844	AHIRMAP	14/99	4.15	0.006
8	0002227	IIRM	7/27	9.37	0.009
17	0045087	IIR	12/485	0.37	0.010
17	0061844	AHIRMAP	17/99	2.99	0.036
12	0061760	AIIR	6/18	8.62	0.045

TCA, T cell activation involved in immune response; NKCA, natural killer cell activation involved in immune response; IRRSP, immune response-regulating signaling pathway; HIR, humoral immune response; PRIR, positive regulation of immune response; RISP, regulation of immune system process; IIR, innate immune response; AIR, adaptive immune response; AHIRMAP, antimicrobial humoral immune response mediated by antimicrobial peptide; IIRM, innate immune response in mucosa; AIIR, antifungal innate immune response.

## Candidate X genes in lupus

X chromosome genes that escape X inactivation; and, thus have expression of the gene from each of X chromosome, are candidates to mediate the X chromosome dose effect. Our attention was drawn to two genes in the toll like receptor 7 (TLR7) pathway that routinely escape X inactivation; namely, *TLR7* itself and *TASL* (TLR Adaptor Interacting With Endolysosomal SLC15A4). The TLR7 pathway is critical for the pathogenesis of SLE, both in murine models and humans. For instance, rare gain-of-function TLR7 mutations can cause monogenic pediatric SLE (27–29) and mice with TLR7 over-expression due to a translocation between the X and Y chromosome develop a lupus-like illness (30, 31). The TLR7 protein is localized to the endosome and is critical for recognition of viruses and subsequent activation of the innate immune system. TLR7 binds single-stranded RNA or metabolites thereof, which activates the pathway, leading to production of interferon as well as other cytokines (32). Furthermore, common population variants of genes encoding protein that function in the TLR7 pathway show genetic association to the SLE phenotype. These include TLR7, TASL, SLC15a4 (a binding partner of TASL (33)), and UNC93B1, a regulator of TLR7 movement into the endosome (34–37). Many functional studies also implicate the TLR7 pathway in SLE pathogenesis in both human and murine lupus models (30, 31, 38–43).

Given the critical nature of the TLR7 pathway in SLE and the association of X chromosome number with the sex bias of the disease, we elected to study the role of TASL in the TLR7 pathway. As described above, the TASL gene routinely escapes X inactivation and TASL is expressed in several immune cells, including B lymphocytes and monocytes, contains an SLE risk allele (19, 35) and binds SLC15A4 on the lysosomal surface (44). SLC15a4 regulates lysosomal pH, to which TLR7 signaling is highly sensitive (45, 46). In addition, knockout of the gene is known to abrogate TLR7 signaling (47).

Given these data, we undertook studies to examine the role of TASL in the TLR7 pathway (48). In particular, since TASL and SLC15a4 are binding partners and SLC15a4, at least in part, determines lysosomal pH, we studied lysosomal pH. First, we examined expression of the TASL protein in human primary monocytes, B cells and lymphoblastoid cells lines. In each case, TASL was expressed more highly in female cells compared to male cells (49). Additional studies from Odham et al, also found TASL was more highly expressed in female cells and this sexual dimorphism was magnified when stimulated with type I interferons (50). Using a ratiometric measurement of lysosomal pH via fluorescence in unstimulated female monocytes, we found lysosomal pH averaged 4.9 versus 5.6 in male cells (p=0.0001) (48). A similar difference in lysosomal pH was also found between male and female B cells and dendritic cells, while we did not find a female:male dichotomy for lysosomal pH in NK or T cells, neither of which express TASL (48). Thus, the sex difference in lysosomal pH is likely to be associated with increased TLR7 signaling, and may be dependent upon increased expression of TASL in female cells.

In order to determine if, in fact, TASL participates in lysosomal pH regulation and TLR7 signaling, we undertook a series of knockdown experiments using CRISPR-Cas9 and primary human monocytes (CD14+/CD16−). In female cells treated with a TLR7 agonist, TASL knockdown abrogated interferon-alpha, IL-6 and TNF production (49). Thus, TASL is critical for TLR7 pathway signaling. Furthermore, knockdown of TASL expression resulted in a rise in lysosomal pH in female monocytes to the pH we found in male monocytes. And, intracellular transport of NOD1 antigens, a function of SLC15a4, was also abrogated by TASL knockdown (49). However, it should be noted that these results have not been independently replicated; and, thus, are not confirmed.

Several other lines of evidence support a sex-biased function of the TRL7 pathway (51–53). Our studies in primary monocytes and LCLs suggest TASL is involved in the TLR7 in a sexually dimorphic manner such that lysosomal pH is lower and TLR7 signaling greater in female versus male cells. As of late, studies on TASL have shown that the once uncharacterized protein functions as enzyme that regulates interferon regulatory factor 5 (IRF5), colocalizes with TLR7 and is interferon inducible. TASL ability to increase interferon production (our work and others) and its own protein level to be subsequently amplified by interferon stimulations suggest a positive feedforward response that would result in increased production that is often found in SLE affected subjects. Thus, increased expression of both TLR7 (54) and TASL (48, 49) may underlie not only improved outcome of women compared to men in some infections (55) but also female disposition to autoimmunity mediated via TLR7 (56).

## XIST in lupus

Other investigators have taken a different tack in studying the role of the X chromosome in the sex bias of SLE (57, 58); however, the data generated also concern the TLR7 pathway. As mentioned above, XIST long non-coding RNA mediates X chromosome inactivation (**Figure 1**); and, thus, is expressed only in cells with more than one X chromosome. Dou and colleagues preformed a series of experiments that indicate XIST is a source of ligand for TLR7; and, of course, this is a sex specific source of ligand (57, 58).

First, these investigators noted that XIST is rich in potential TLR7 ligands. A putative TLR7 stimulatory motif, the UU dinucleotide, was found 2,140 times in XIST RNA. XIST was the sex-biased transcript with the highest degree of UU dinucleotide gene expression; and, further, was the only sex-biased expression source of the extended TLR7 motif 5′-GUCCUUCAA-3′ (57, 58). Overall, XIST was the strongest sex biased source of self TLR7 ligand.

Next, these investigators turned to stimulation of TLR7 by XIST nucleotides using HEK-hTLR7 cells as a reporter. The extended TLR7 motif found in XIST as well as a longer sequence of XIST (containing the A-repeat, UU dinucleotide rich region) were also found to stimulate TLR7 signaling as indicated by production of interferon-alpha. Further, not only was the response due to specific binding of XIST nucleotide and dose-dependent, the TLR7 response was inhibited by depletion of XIST as well by hydroxychloroquine (57). Additional studies found that XIST levels were higher in peripheral leukocytes among women with SLE compared to non-SLE affected matched controls, and that levels of XIST correlated with disease activity. The investigators concluded, and we certainly agree, that the XIST long non-coding RNA is the most potent source of sex biased TLR7 ligands in female cells.

## XIST, TLR7, TASL in lupus and other autoimmune diseases – an hypothesis

We further conclude that these two sets of data suggest synergism for a female biased expansion of the TLR7 signaling pathway that could underlie the X chromosome dose effect found in various autoimmune diseases, including SLE (12–15), Sjögren’s disease (13, 16), polymyositis/dermatomyositis (17), and systemic sclerosis (17). The idea, we think, is straight forward. XIST RNA supplies TLR7 ligand in female cells. In addition, female B lymphocytes, dendritic cells, and monocytes have enhanced TLR7 pathway signaling by virtue of the over-expression (compared to male cells) of not only TLR7 but also TASL. Enhanced TLR7 signaling activity deploys a feed forward loop in the TLR7 pathway that leads to increased expression and activity of the pathway (59). Thus, both increased ligand and enhanced activity support further enhancement of TLR7 signaling in female cells. Of course, these phenomena are universal in cells with more than one X chromosome; that is, from women or Klinefelter men. So, other factors must be in play such as other genetics or environmental exposure.

## TLR7 signaling and environmental triggers in lupus

What environmental exposure might interact with this sex-biased enhancement of TLR7 signaling induced by Xist and genes in the TLR7 pathway that escape X inactivation? One candidate is Epstein Barr virus (EBV). Epidemiological evidence supports the idea that this near ubiquitous infection is necessary but not sufficient for the expression of SLE as well as multiple sclerosis, and there some evidence in Sjögren’s disease (60–65). Recent studies have found that single nucleotide polymorphisms demonstrating genetic association with SLE or Sjögren’s disease are more likely to be found in promoter regions bound by the EBV transcription factor EBV nuclear antigen 2 (EBNA2) (66, 67). Overall, the preponderance of evidence indicates that EBV infection is likely one of the environmental triggers for disease. Furthermore, EBV infects B lymphocytes, a cell type with expression of TASL, engaging and increasing expression of TLR7 (68). B cell hyperplasia is one of the hallmarks of systemic autoimmune disease (69). Thus, these data concerning enhanced expressed XIST, TLR7 and TASL in female cells impacting TLR7 signaling may interact with data concerning a role of EBV in promoting SLE and other autoimmune diseases (62, 63, 70). Of course, estrogen and differential expression of estrogen-regulated genes remain a potential biological trigger of the disease. The sex bias of SLE is present in prepubescent children at about 5 to 1, but of course is less pronounced than after puberty (71). These data suggest an effect of estrogen. Further, there are clear effects of estrogen on B lymphocytes and humeral immunity (72, 73) with effects on development, immune tolerance, immunoglobulin somatic hypermutation, and class switching. In addition, some estrogen effects in B cells may be mediated through cell surface (as opposed to nuclear) estrogen receptors (74).

## Summary

**Figure 2:**
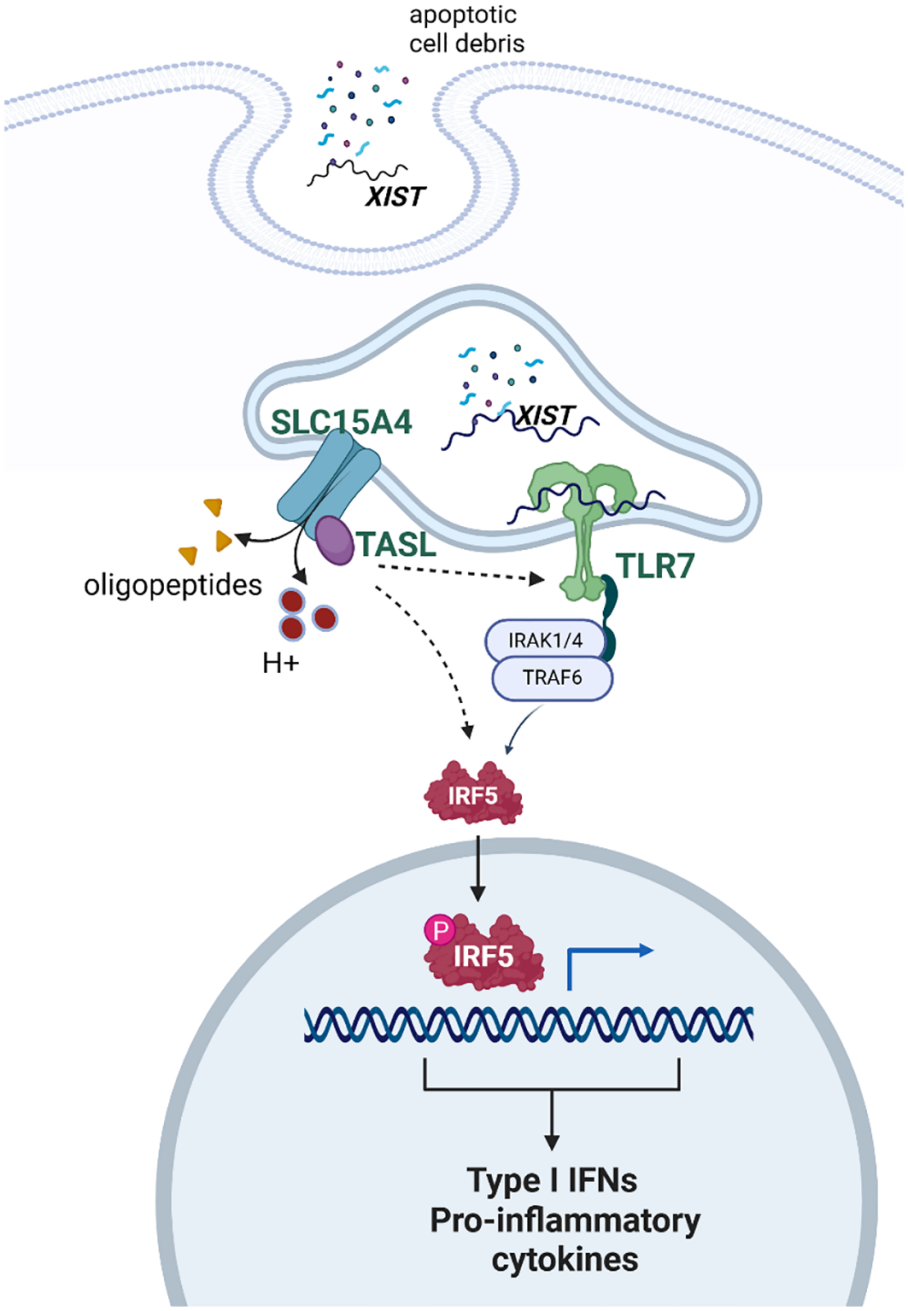
Schematic depicting the proposed interaction of XIST, TLR7, and TASL in response to self-antigen. XIST provides ligand for TLR7. Once TLR7 signaling is activated, there is a feed forward stimulation of the pathway. The genes for both TLR7 and TASL are on the X chromosome and escape X inactivation. Thus, some data suggest that TRL7 signaling is more robust in female cells, compared to male cells, on this basis. Created with Biorender.com.

The evidence is strong that the number of X chromosomes is important for the female bias of some, but not all, autoimmune diseases. The mechanism by which a dose effect for the X chromosome is not understood. Available evidence suggests that multiple factors may play roles that are complementary. These include expression of XIST, which provides TRL7 ligand, and escape of X inactivation by genes whose protein products are critical for TLR7 signaling (see **Figure 2**).
