# Supplementary material for: The toll like receptor 7 pathway and the sex bias of systemic lupus erythematosus
Source: Front Immunol. 2025 Feb 20;16:1479814. doi: 10.3389/fimmu.2025.1479814 (PMC11882868; doi:10.3389/fimmu.2025.1479814)
Supplement: Supplementary file 1 [file Table1.pdf]

Supplementary Table 1. GO categories in which there was enrichment on the X chromosome.

GO category/ID	#/total	OR	FDR p value
miRNA-mediated post-transcriptional gene silencing/0035195	57/574	3.03	5.39E-09
nucleus/0005634	289/5649	1.52	3.80E-07
negative regulation of transcription by RNA polymerase II/0000122	70/916	2.28	1.79E-06
mRNA base-pairing translational repressor activity/1903231	30/297	3.08	0.000148578
histone deacetylase binding/0042826	18/128	4.48	0.000281934
RISC complex/0016442	36/411	2.63	0.000332703
detection of chemical stimulus involved in sensory perception of smell/0050911	1/405	0.07	0.002357803
tRNA threonylcarbamoyladenosine metabolic process/0070525	4/5	109.36	0.002366519
olfactory receptor activity/0004984	1/403	0.07	0.002484309
nuclear RNA export factor complex/0042272	4/6	54.68	0.005716037
protein binding/0005515	531/12712	1.26	0.010413196
methylated histone binding/0035064	11/74	4.78	0.012942746
arylsulfatase activity/0004065	5/14	15.19	0.01799862
Rab protein signal transduction/0032482	5/15	13.67	0.024225817
neurexin family protein binding/0042043	5/15	13.67	0.024225817
mRNA 3'-UTR binding/0003730	21/242	2.60	0.029362988
